# Current Progress in Biopolymer-Based Bionanocomposites and Hybrid Materials

**DOI:** 10.3390/polym14173479

**Published:** 2022-08-25

**Authors:** R. A. Ilyas, S. M. Sapuan, Emin Bayraktar

**Affiliations:** 1 School of Chemical and Energy Engineering, Faculty of Engineering, Universiti Teknologi Malaysia (UTM), Johor Bahru 81310, Malaysia; 2 Centre for Advanced Composite Materials, Universiti Teknologi Malaysia (UTM), Johor Bahru 81310, Malaysia; 3 Institute of Tropical Forest and Forest Products (INTROP), Universiti Putra Malaysia, Serdang 43400, Malaysia; 4 Advanced Engineering Materials and Composites, Department of Mechanical and Manufacturing Engineering, Faculty of Engineering, Universiti Putra Malaysia, Serdang 43400, Malaysia; 5 School of Mechanical and Manufacturing Engineering, ISAE-SUPMECA Institute of Mechanics of Paris, 93400 Saint-Ouen, France

*Current Progress in Biopolymer-Based Bionanocomposites and Hybrid Materials* is a newly opened Special Issue of Polymers, which aims to publish original and review papers on the new scientific and applied research and make boundless contributions to the findings and understanding of the reinforcing effects of various synthetic and natural fibres on the performance of biopolymer composites. This Special Issue also covers the hybrid nanofibre-reinforced biopolymer nanocomposites’ fundamentals, characterisation, and applications.

In recent years, the development of biopolymers based on constituents obtained from natural resources has been gaining much attention [1,2]. The exploitation of biopolymers to engineer advanced bionanocomposites and hybrid materials is the focus of increasing scientific activity, explained by the growing environmental concerns and the interest in the novel features and multiple functionalities of these macromolecules.

Today, nanomaterial-reinforced polymers are used in several applications including in packaging [2,3,4]; electronic, electrical, structural, and energy storage [5]; in automotives [6]; in filter, coating, and bone tissue engineering, and in drug delivery [7], and more. The continuous development and appearance on the market of new high-performance reinforcing nanomaterials in polymer composites have constituted a strong challenge for researchers to design and adapt new functional nano-composites for several applications. The term bionanocomposites was introduced several years ago to express an emerging class of bionano- and bionanohybrid materials, resulting from the reinforcement of biopolymers, such as proteins (gelatin, casein, soy, and gluten), polysaccharides (cellulose, starch, chitosan, pectin, alginate, carrageenan, and glycogen), lipids (cutin), and nucleic acids with inorganic or organic solids at the nanoscale [8,9,10,11,12,13]. Such organic fractions comprise nanocrystalline cellulose [14,15,16], nanofibrillated cellulose [17,18] (Figure 1), bacterial nanocellulose [19], and lignin nanoparticles [20], whereas inorganic fractions consist of finely divided solids, spanning from clays to phosphates or carbonates, whose origins can be either synthetic or natural.

As will become increasingly clear for the reader throughout the collection of authoritative research and reviews in this Special Issue, the relevance of coupling biopolymers with organic and inorganic fillers, through innovative architectures, is twofold. First, it contradicts the idea that biopolymers are either eco-friendly or high-performance. Second, it demonstrates how the properties resulting from these biopolymers are highly significant in applications such as food packaging, water treatment, gas-diffusion barriers, electronic devices, agriculture, sensing devices, flame retardancy, automotive parts, adhesives, regenerative medicine, tissue engineering, and drug delivery [21].

In this Special Issue, we aim to capture the cutting edge of the state of the art in research pertaining to biopolymer-based bionanocomposites and hybrid materials and their advanced applications. Contributions to the processing of biopolymers and bionanocomposites, the use of diverse biopolymer sources such as polysaccharides, the reinforcement of nanosized materials with biopolymers, and applications of these biopolymers, bionanocomposites, and biohybrid materials will constitute the backbone of this Special Issue.

## Figures and Tables

**Figure 1:**
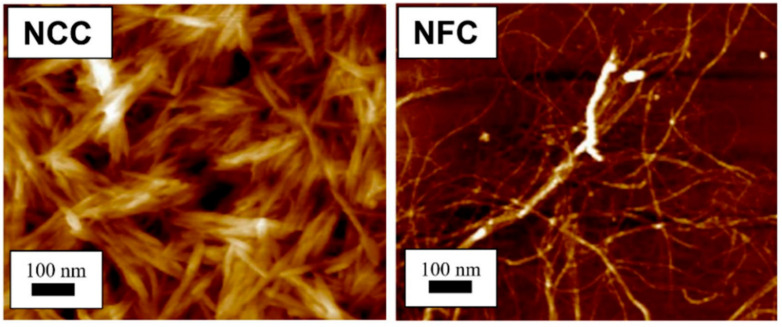
Atomic force microscopy images show different structures between nanocrystalline cellulose (NCC) and nanofibrillated cellulose (NFC).

## References

[1] Tarique J., Sapuan S.M., Khalina A., Ilyas R.A., Zainudin E.S. (2022). Thermal, flammability, and antimicrobial properties of arrowroot (Maranta arundinacea) fiber reinforced arrowroot starch biopolymer composites for food packaging applications. Int. J. Biol. Macromol..

[2] Nazrin A., Sapuan S.M., Zuhri M.Y.M., Tawakkal I.S.M.A., Ilyas R.A. (2022). Flammability and physical stability of sugar palm crystalline nanocellulose reinforced thermoplastic sugar palm starch/poly (lactic acid) blend bionanocomposites. Nanotechnol. Rev..

[3] Rozilah A., Jaafar C.N.A., Sapuan S.M., Zainol I., Ilyas R.A. (2020). The Effects of Silver Nanoparticles Compositions on the Mechanical, Physiochemical, Antibacterial, and Morphology Properties of Sugar Palm Starch Biocomposites for Antibacterial Coating. Polymers.

[4] Alias A.H., Norizan M.N., Sabaruddin F.A., Asyraf M.R.M., Norrrahim M.N.F., Ilyas A.R., Kuzmin A.M., Rayung M., Shazleen S.S., Nazrin A. (2021). Hybridization of MMT/Lignocellulosic Fiber Reinforced Polymer Nanocomposites for Structural Applications: A Review. Coatings.

[5] Nurazzi N.M., Sabaruddin F.A., Harussani M.M., Kamarudin S.H., Rayung M., Asyraf M.R.M., Aisyah H.A., Norrrahim M.N.F., Ilyas R.A., Abdullah N. (2021). Mechanical Performance and Applications of CNTs Reinforced Polymer Composites—A Review. Nanomaterials.

[6] Nurazzi N.M., Asyraf M.R.M., Rayung M., Norrrahim M.N.F., Shazleen S.S., Rani M.S.A., Shafi A.R., Aisyah H.A., Radzi M.H.M., Sabaruddin F.A. (2021). Thermogravimetric Analysis Properties of Cellulosic Natural Fiber Polymer Composites: A Review on Influence of Chemical Treatments. Polymers.

[7] Sharma S., Sudhakara P., Singh J., Ilyas R.A., Asyraf M.R.M., Razman M.R. (2021). Critical Review of Biodegradable and Bioactive Polymer Composites for Bone Tissue Engineering and Drug Delivery Applications. Polymers.

[8] Ilyas R.A., Zuhri M.Y.M., Aisyah H.A., Asyraf M.R.M., Hassan S.A., Zainudin E.S., Sapuan S.M., Sharma S., Bangar S.P., Jumaidin R. (2022). Natural Fiber-Reinforced Polylactic Acid, Polylactic Acid Blends and Their Composites for Advanced Applications. Polymers.

[9] Norfarhana A.S., Ilyas R.A., Ngadi N. (2022). A review of nanocellulose adsorptive membrane as multifunctional wastewater treatment. Carbohydr. Polym..

[10] Ilyas R.A., Zuhri M.Y.M., Norrrahim M.N.F., Misenan M.S.M., Jenol M.A., Samsudin S.A., Nurazzi N.M., Asyraf M.R.M., Supian A.B.M., Bangar S.P. (2022). Natural Fiber-Reinforced Polycaprolactone Green and Hybrid Biocomposites for Various Advanced Applications. Polymers.

[11] Ilyas R.A., Aisyah H.A., Nordin A.H., Ngadi N., Zuhri M.Y.M., Asyraf M.R.M., Sapuan S.M., Zainudin E.S., Sharma S., Abral H. (2022). Natural-Fiber-Reinforced Chitosan, Chitosan Blends and Their Nanocomposites for Various Advanced Applications. Polymers.

[12] Nabilah Haris N.I., Hassan M.Z., Ilyas R.A., Suhot M.A., Sapuan S.M., Dolah R., Mohammad R., Asyraf M.R.M. (2022). Dynamic mechanical properties of natural fiber reinforced hybrid polymer composites: A review. J. Mater. Res. Technol..

[13] Ilyas R.A., Sapuan S.M., Asyraf M.R.M., Dayana D.A.Z.N., Amelia J.J.N., Rani M.S.A., Norrrahim M.N.F., Nurazzi N.M., Aisyah H.A., Sharma S. (2021). Polymer Composites Filled with Metal Derivatives: A Review of Flame Retardants. Polymers.

[14] Ilyas R.A., Sapuan S.M., Atikah M.S.N., Asyraf M.R.M., Rafiqah S.A., Aisyah H.A., Nurazzi N.M., Norrrahim M.N.F. (2021). Effect of hydrolysis time on the morphological, physical, chemical, and thermal behavior of sugar palm nanocrystalline cellulose (Arenga pinnata (Wurmb.) Merr). Text. Res. J..

[15] Abral H., Ariksa J., Mahardika M., Handayani D., Aminah I., Sandrawati N., Pratama A.B., Fajri N., Sapuan S.M., Ilyas R.A. (2020). Transparent and antimicrobial cellulose film from ginger nanofiber. Food Hydrocoll..

[16] Sabaruddin F.A., Paridah M.T., Sapuan S.M., Ilyas R.A., Lee S.H., Abdan K., Mazlan N., Roseley A.S.M., Abdul Khalil H.P.S. (2020). The effects of unbleached and bleached nanocellulose on the thermal and flammability of polypropylene-reinforced kenaf core hybrid polymer bionanocomposites. Polymers.

[17] Ilyas R.A., Sapuan S.M., Ishak M.R., Zainudin E.S. (2019). Sugar palm nanofibrillated cellulose (Arenga pinnata (Wurmb.) Merr): Effect of cycles on their yield, physic-chemical, morphological and thermal behavior. Int. J. Biol. Macromol..

[18] Syafri E., Sari N.H., Mahardika M., Amanda P., Ilyas R.A. (2022). Isolation and characterization of cellulose nanofibers from Agave gigantea by chemical-mechanical treatment. Int. J. Biol. Macromol..

[19] Abral H., Chairani M.K., Rizki M.D., Mahardika M., Handayani D., Sugiarti E., Muslimin A.N., Sapuan S.M., Ilyas R.A. (2021). Characterization of compressed bacterial cellulose nanopaper film after exposure to dry and humid conditions. J. Mater. Res. Technol..

[20] Trevisan H., Rezende C.A. (2020). Pure, stable and highly antioxidant lignin nanoparticles from elephant grass. Ind. Crops Prod..

[21] Mohd Nurazzi N., Asyraf M.R.M., Khalina A., Abdullah N., Sabaruddin F.A., Kamarudin S.H., Ahmad S., Mahat A.M., Lee C.L., Aisyah H.A. (2021). Fabrication, Functionalization, and Application of Carbon Nanotube-Reinforced Polymer Composite: An Overview. Polymers.

